# Characterization of double humanized BLT-mice with stable engraftment of a human gut bacterial microbiome

**DOI:** 10.3389/frmbi.2024.1404353

**Published:** 2024-07-04

**Authors:** Lance Daharsh, Saroj Chandra Lohani, Amanda E. Ramer-Tait, Qingsheng Li

**Affiliations:** ^1^ School of Biological Sciences, University of Nebraska-Lincoln, Lincoln, NE, United States; ^2^ Nebraska Center for Virology, University of Nebraska-Lincoln, Lincoln, NE, United States; ^3^ Department of Food Science and Technology, University of Nebraska-Lincoln, Lincoln, NE, United States; ^4^ Nebraska Food for Health Center, University of Nebraska-Lincoln, Lincoln, NE, United States

**Keywords:** double humanized mice, stable engraftment, human gut microbiota, mouse model, BLT mice

## Abstract

Humanized mice with human-like immune systems are commonly used to study immune responses to human-specific pathogens. However, one limitation of using humanized mice is their native murine gut microbiota, which significantly differs from that in humans. Given the importance of the gut microbiome to human health, these differences may profoundly impact the ability to translate results from humanized mouse studies to humans. Further, there is a critical need for improved pre-clinical models to study the complex *in vivo* relationships of the gut microbiome, immune system, and human disease. We previously created double humanized mice with a functional human immune system and a stable, human-like gut microbiome. Here, we characterized the engrafted human gut bacterial microbiome in our double humanized mouse model generated by transplanting fecal material from healthy human donors into the gut of humanized mice. Analysis of bacterial microbiomes in fecal samples from double humanized mice revealed they had unique 16S rRNA gene profiles consistent with those of the individual human donor samples. Importantly, transplanted human-like gut microbiomes were stable in mice for the duration of the study, extending up to 14.5 weeks post-transplant. Microbiomes of double humanized mice also harbored predicted functional capacities that more closely resembled those of the human donors than humanized mice. In conclusion, our study highlights the successful engraftment of human fecal microbiota in BLT humanized mice and underscores the stability of this model, offering a valuable platform for investigating the intricate interplay among the human gut microbiome, immune system, and various diseases *in vivo*.

## 1 Introduction

The complex ecosystem of the gut microbiome plays a critical role in human health and disease (Turnbaugh et al., 2006; Qin et al., 2010; Clemente et al., 2018; Gopalakrishnan et al., 2018; Routy et al., 2018). Specifically, the gut microbiome has a highly reciprocal and dynamic relationship with the immune system. Antigens derived from the gut microbiome influence host immune responses, and the immune system, in turn, contributes to shaping the spatial distribution and composition of the gut microbiota (Kau et al., 2011; Hooper et al., 2012; Maynard et al., 2012). Humanized mice (hu-mice) with an engrafted human immune system have facilitated important advancements in the study of human cancer, autoimmune diseases, hematopoiesis, and infectious diseases (Sun et al., 2007; Simpson-Abelson et al., 2008; Bankert et al., 2011; Whitfield-Larry et al., 2011; Vudattu et al., 2014; Wang et al., 2014; Stary et al., 2015; Ernst, 2016; Yi et al., 2017). However, gut microbiomes of hu-mice are murine in origin and are often not well-characterized in translational studies. The murine gut microbiome differs substantially in composition and function from that of humans (Xiao et al., 2015), primarily due to anatomical or evolutionary differences as well as other factors such as diet (Nguyen et al., 2015). Considering the importance of the gut microbiota in proper immunological development and influencing immune responses, the murine origin of the microbiome harbored by hu-mice could affect translational study outcomes. Consequently, a need exists to not only characterize the gut microbiomes of hu-mice but also impart these mice with a more human-like gut microbiome to improve the translational aspects of the model for human medicine.

The creation of a new, pre-clinical hu-mice model to study the human immune system in the context of a human microbiome offers numerous benefits over existing options. Many aspects of human disease are difficult or impossible to study directly in humans due to practical or ethical concerns. Non-human primate models are informative; however, they are often resource and cost prohibitive. Many important discoveries in the microbiome field have been made using mouse models; however, translating results from mouse studies to humans has often proven difficult. The use of germ-free mice reconstituted with human-like gut microbiomes has been the gold standard in studying the relationship of the gut microbiome to human health and disease (Turnbaugh et al., 2009; Kennedy et al., 2018). However, working with or deriving germ-free animals requires expertise and facilities that are not always available. Further, many immunodeficient mouse strains commonly used to reconstitute a human immune system, such as NOD.*Cg-Prkdc^scid^Il2rg^tm1Wjl^*/SzJ (NSG) are currently not commercially available as germ-free. We previously created a double hu-mice model featuring a functional human immune system and a human-like gut microbiome under specific pathogen free (SPF) conditions (Daharsh L et al., 2019). Here, we characterized the gut microbiome of the double hu-mice, demonstrating their unique 16S rRNA gene profiles based on the individual human donor sample with which they were colonized. Importantly, the transplanted human-like microbiome was stable in the mice for the duration of the study, up to 14.5 weeks post-transplant. Double hu-mice also harbored gut microbiomes with a more human-like predicted functional capacity compared to their hu-mice counterparts.

## 2 Methods

### 2.1 Generation of hu-mice

All methods described here were conducted as we previously reported (Li et al., 2015; Destache et al., 2016; Yuan et al., 2016; Daharsh L et al., 2019) in accordance with Institutional Animal Care and Research Committee (IACUC)-approved protocols at the University of Nebraska-Lincoln (UNL). Briefly, 6- to 8-week-old female NSG mice (NOD.*Cg-Prkdc^scid^Il2rg^tm1Wjl^*/SzJ, Cat# 005557; Jackson Laboratory, Bar Harbor, ME) were housed and maintained in individual microisolator cages in a rack system designed to regulate air circulation through pre-filters and HEPA filters and fed with irradiated Teklad global 14% protein rodent chow (Teklad 2914; Inotiv, Madison, WI) along with autoclaved acidified drinking water. The second cohort of mice (**Table 1**) was supplemented with a high-calorie gel (DietGel Boost). Prior to surgery to humanize the mouse immune system, mice were subjected to total-body irradiation with a dose of 12 cGy per gram of body weight using the RS200 X-ray irradiator (RAD Source Technologies, Inc., GA). Following irradiation, mice were implanted with a single human fetal thymic tissue fragment sandwiched between two human fetal liver tissue fragments within the left renal capsule. Human CD34+ hematopoietic stem cells were isolated from human fetal liver and injected into mice via tail vein at a range of 1.5-5 × 10^5^ within a 6-hour window following surgery. The human fetal liver tissues and thymus used in this study were obtained from Advanced Bioscience Resources (Alameda, CA). After 9 to 12 weeks, human immune cell reconstitution in peripheral blood was measured by a fluorescence-activated cell sorter (FACS) Aria II flow cytometer (BD Biosciences, San Jose, CA) using antibodies against mCD45-APC, hCD45-FITC, hCD3-PE, hCD19-PE/Cy5, hCD4-Alexa 700, and hCD8-APC-Cy7 (Cat# 103111, 304006, 300408, 302209, 300526, and 301016, respectively; BioLegend, San Diego, CA). Raw data were analyzed with FlowJo (version 10.0; FlowJo LLC, Ashland, OR). All hu-mice used in this study had human immune cell reconstitution with an average of 85% hCD45+ cells in peripheral blood 12 weeks post-surgery. The assignment of mice into the experimental groups was done randomly, ensuring comparable levels of human immune cell reconstitution across the groups.

**Table 1:** Experimental summary of double hu-mice cohorts.

Cohort	Antibiotic start	Antibiotic duration	Number of fecal transplants	Human fecal donor	Maximum weeks post-transplant	Double hu-mice	Double hu-mice fecal samples	Hu-mice	Hu-mice fecal samples
Antibiotic Pilot	After immune reconstitution	9 days	0	NA	0	0	0	6	9
Donor 65 Cohort 1	After immune reconstitution	14 days	2 (24 & 48 hr post antibiotics)	65	13.5	3	35#	3	23
Donor 65 Cohort 2	After immune reconstitution	14 days	2 (24 & 48 hr post antibiotics)	65	14.5	2	16#	8	8
Donor 74	After immune reconstitution	7 days	2 (24 & 48 hr post antibiotics)	74	6	3	17#	3*	3*
Donor 82	After immune reconstitution	7 days	2 (24 & 48 hr post antibiotics)	82	6	4	27#	3*	3*
Donor Mix	3 days after hu-mice surgery	14 days	2 (24 & 48 hr post antibiotics)	Equal mixture of 65, 74, & 82	9	4	21#	9	10

#Includes 1 pre-fecal transplant sample for each of the double hu-mice.

*Control hu-mice were the same for Donor 74 and Donor 82 groups.

### 2.2 Generation of double hu-mice

The hu-mice generated described above in section 2.1 by the bone-marrow, liver, thymus (BLT) method of humanization was then used to create double hu-mice with both the human immune system and human microbiota based on antibiotic-induced microbiota depletion procedure followed by oral gavage of human fecal microbiota.

#### 2.2.1 Antibiotic treatment

A cocktail of broad-spectrum antibiotics consisting of Ampicillin (1 g/L), Metronidazole (1 g/L), Neomycin (1 g/L), and Vancomycin (0.5 g/L) was prepared fresh daily and given to the mice ad libitum in the drinking water along with grape-flavored Kool-Aid to improve palatability. Mice in the control group were provided solely with grape flavored Kool-Aid in the drinking water prepared fresh daily. During antibiotic treatment, cages were replaced daily to limit re-inoculation of pre-existing bacteria in the mice due to their coprophagic behavior. Antibiotics were given for 14 days for double hu-mice engrafted with Donor 65 and Donor Mix and for seven days for double hu-mice engrafted with Donor 74 and Donor 82. Mice in the pilot study were given antibiotics via oral gavage as follows: For three days, mice received anti-fungal Amphotericin B treatment (1 mg/kg) twice daily via oral gavage and were then given the antibiotic cocktail along with Amphotericin B twice daily via oral gavage. After four days of treatment, the Amphotericin B was stopped due to toxicity concerns, and after 10 days of treatment, oral gavage was reduced to once daily. Post-antibiotic treatment, all mice were given autoclaved non-acidified deionized drinking water.

During the first few days of antibiotic treatment, mice lost a considerable amount of body weight (10–20%). The weight loss plateaued after three to four days and remained steady for the remainder of antibiotic treatment. Throughout antibiotic treatment, we closely monitored the body weight of the mice, and when necessary, administered intraperitoneal (IP) injection of Ringer’s solution to alleviate any effects of dehydration. After fecal transplant, the mice regained weight and returned to their pre-existing weight within two weeks post-transplant. During antibiotic treatment, there was a large reduction in spleen size and a large increase in cecum size compared to controls. This is similar to the morphology observed in germ-free mice, providing further evidence for the efficacy of the antibiotic regimen (Smith et al., 2007).

#### 2.2.2 Donor samples and fecal microbiota transplant

Three unique microbiota preparations (Donor 65, Donor 74, and Donor 82) were procured from OpenBiome (Massachusetts, USA). An unbiased human donor sample (Donor mix) was prepared within an anaerobic chamber by mixing an equal portion of the three different donor microbiota preparations. Hu-mice were grouped into multiple cohorts and 200 μL of human fecal material from one of three unique healthy human donors or an equal mixture of all three (Donor mix) were given via oral gavage at 24 and 48 hours after the completion of antibiotic pre-treatment. 16S rRNA sequencing data on the three donors was also supplied by OpenBiome.

### 2.3 Mouse fecal collection and DNA extraction

Fresh fecal samples from the mice were collected within a biosafety hood by placing individual mice into autoclaved paper bags. Fecal samples were stored at −80°C in 1.5 mL Eppendorf tubes until DNA extraction. DNA was extracted from the fecal samples using the method described previously (Martinez et al., 2009). Briefly, fecal samples were washed three times with 1 mL PBS buffer (pH 7), followed by incubation at 60° C for 30 minutes along with 750 μL of lysis buffer, 300 mg of autoclaved 0.1 mm zirconia/silica beads (Biospec), 85 μL of 10% SDS solution, and 40 μL of Proteinase K (15mg/mL, Promega, Cat# MC500B). After incubation, 500 μL of phenol:chloroform:isoamyl alcohol (25:24:1) was added, and cells were physically lysed using a bead beater (BioSpec Mini-Beadbeater-16) for 2 minutes. The upper phase of the sample was collected and additional 500 μL of phenol:chloroform:isoamyl alcohol (25:24:1) was added and spun down to separate upper phase containing the DNA, which was further purified twice with 500 μL of chloroform:isoamyl alcohol (24:1). DNA was then precipitated overnight at −20°C with 100% Ethanol (2.5 x volume of sample) and 3M sodium acetate (0.1 x volume of sample) and dried at room temperature. Dried DNA was resuspended in 100 μL of Tris-Buffer (10mM, pH8) and stored at −20°C for future use.

### 2.4 16S rRNA bacterial gene sequencing

16S rRNA bacterial gene sequencing was performed at the University of Nebraska Medical Center Genomics Core Facility using Illumina MiSeq. DNA normalization and library prep were performed, followed by V3-V4 16S rRNA amplicon gene sequencing using a MiSeqV2 (Illumina). The following primer sequences were used: (Primer sequences: Forward Primer = 5’

TCGTCGGCAGCGTCAGATGTGTATAAGAGACAGCCTACGGGNGGCWGCAG 16S Amplicon PCR Reverse Primer = 5’ GTCTCGTGGGCTCGGAGATGTGTATAAGAGACAGGACTACHVGGGTATCTAATCC

Illumina overhangs: Forward overhang: 5’ TCGTCGGCAGCGTCAGATGTGTATAAGAGACAG‐[locusspecific sequence] Reverse overhang: 5’ GTCTCGTGGGCTCGGAGATGTGTATAAGAGACAG‐[locusspecific sequence]).

### 2.5 Generation of the amplicon sequence variant table and data analysis

Illumina-sequenced paired-end fastq files were demultiplexed by sample and barcodes were removed by the sequencing facility. Amplicon sequence variant (ASV) table was generated following the DADA2 v1.8 R package pipeline using the University of Nebraska Holland Computer Center Crane cluster (Callahan et al., 2016). Taxonomy was assigned using the Greengenes 13.8 database and RDP Classifier with a minimal confidence score of 0.80 (Wang et al., 2007; Caporaso et al., 2010). Analysis was performed using R package mctoolsr (https://github.com/leffj/mctoolsr/), and samples were rarified to 13,000 ASVs for downstream analysis. Additional testing of differences between groups was performed using LEfSe (Segata et al., 2011). SourceTracker was used to evaluate the stability of the transferred donor microbiome in the double hu-mice (Knights et al., 2011). GraphPad Prism 5 was used to create some figures. DADA2 generated ASVs were used to predict the functional metagenome capacity using PICRUSt (Langille et al., 2013) via the following pipeline (https://github.com/vmaffei/dada2_to_picrust).

## 3 Results

### 3.1 Gut microbiomes of double hu-mice were distinct and more human-like compared to hu-mice

**Figure 1:**
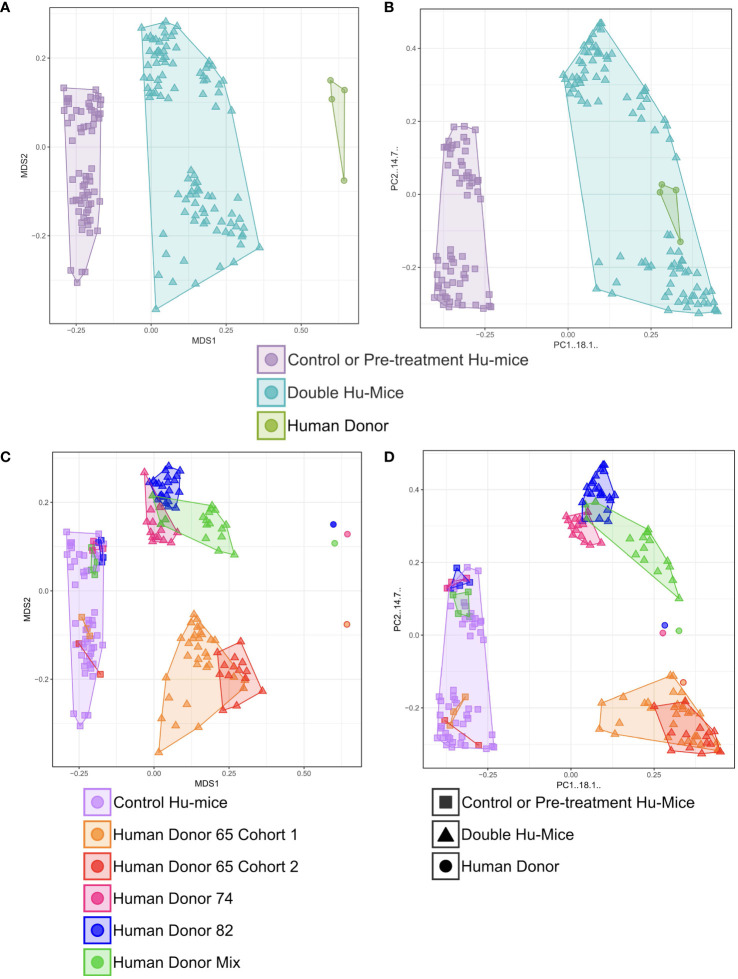
Gut microbiomes of double hu-mice were distinct and more human-like compared to hu-mice and feature donor specific profiles. Double hu-mice clustered distinctly than the human donor samples and pre- treatment or untreated control hu-mice in Non-metric multidimensional scaling (NMDS) plot **(A)** and in Principal coordinate analysis (PCoA) plot **(B)**. Plot displaying human donor specific profiles of the double hu-mice in NMDS plot **(C)** and in PCoA plot **(D)**.

**Figure 2:**
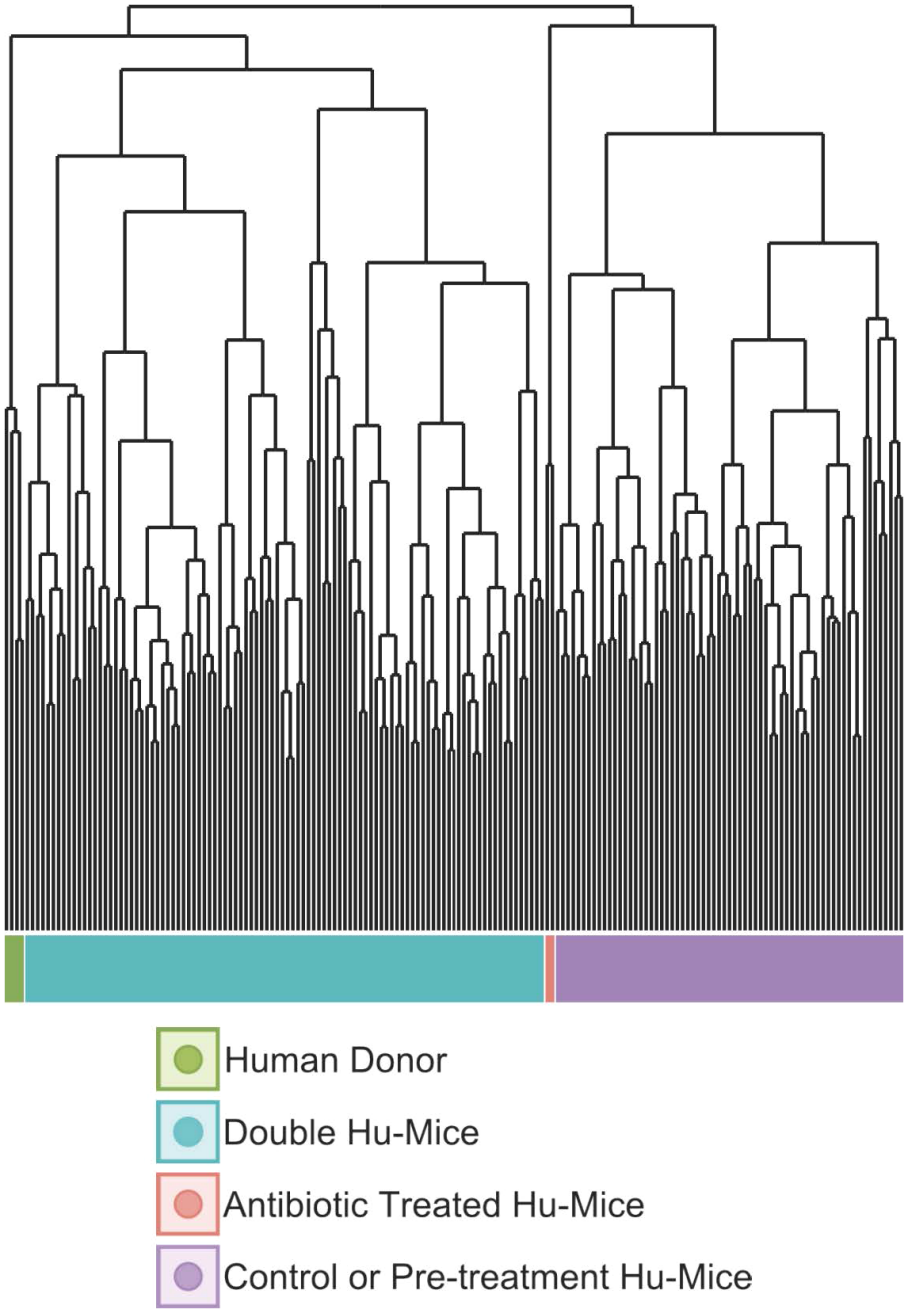
The gut microbiomes of double hu-mice clustered with those of human donor fecal samples. Dendrogram based on Bray-Curtis distances for the gut microbiome profiles of donor fecal samples (Human Donor), double hu-mice (Double Hu-Mice), antibiotic treated hu-mice (Antibiotic Treated Hu-Mice), and untreated control or pre-treatment hu-mice (Control or Pre-treatment Hu-Mice).

Multiple cohorts of double hu-mice were created using fecal material from one of three unique healthy human donors or an equal mixture of all three (**Table 1**). We used 16S rRNA gene sequencing to characterize the gut bacterial microbiome of 100 fecal samples from 16 double hu-mice and compare them with 67 fecal samples representing the pre-existing murine gut microbiomes of hu-mice and the profiles of the four human fecal donor samples. To visualize beta-diversity relationships between hu-mice, double hu-mice, and the human donor samples, non-metric multidimensional scaling (NMDS) and principal coordinate analysis (PCoA) plots were created. In the NMDS plot, the gut microbiome profiles of the three groups separated into distinct clusters (**Figure 1A**). In the PCoA plot, the human donor samples fell within the double hu-mice hull, which was distinct from the hu-mice profiles (**Figure 1B**). Both dimensionality reduction methods revealed that following human gut microbiome engraftment, double hu-mice exhibited a distinct gut microbial profile that clustered closer to the human donor samples when compared to hu-mice harboring murine gut microbiomes. Importantly, there was no reversion to hu-mice profiles post-transplant. Displaying the relatedness of the samples through a hierarchical dendrogram based on Bray-Curtis distances further confirmed the similarity of double hu-mice microbiomes to the human donor samples and differentiated them from those of hu-mice (**Figure 2**).

We also observed that double hu-mice maintained the pre-existing relationships among the microbiome profiles of the human fecal donors (**Figures 1C, D**). Importantly, pre-treatment samples from corresponding double hu-mice in each cohort had similar gut microbiome profiles as untreated control hu-mice. After engraftment, double hu-mice resembled the individual human donor that was transplanted as demonstrated by the relationships between the human donor microbiome profiles. Human donors 74 and 82 had more similar profiles to one another than to human donor 65. This relationship was maintained in the double hu-mice after engraftment. We also prepared an “unbiased” human sample by mixing equal parts of the three human donor fecal samples, designated hereafter as donor mix. The microbiome profile of this mixed sample resembled a mixture of the three individual human donor profiles. Specifically, the mixed donor sample more closely resembled individual donors 74 and 82, and this observation was mirrored in the double hu-mice engrafted with the donor mix sample.

We also investigated the impact of antibiotic treatment duration on the engraftment of the human gut microbiome. The double hu-mice engrafted with human donors 74 and 82 were generated after only seven days of antibiotic pre-treatment. Their microbiome profiles were less similar to the human donor profiles than to those from cohorts pre-treated with antibiotics for 14 days prior to fecal transplant (**Figures 1C, D**). We found the two weeks of antibiotic treatment was optimal for the creation of double hu-mice. Together, these results demonstrate that our approach of generating double hu-mice is reproducible and able to create hu-mice with unique 16S rRNA gene profiles based on the individual human fecal donor.

### 3.2 Gut microbiomes of double hu-mice had increased levels of alpha diversity compared to hu-mice

**Figure 3:**
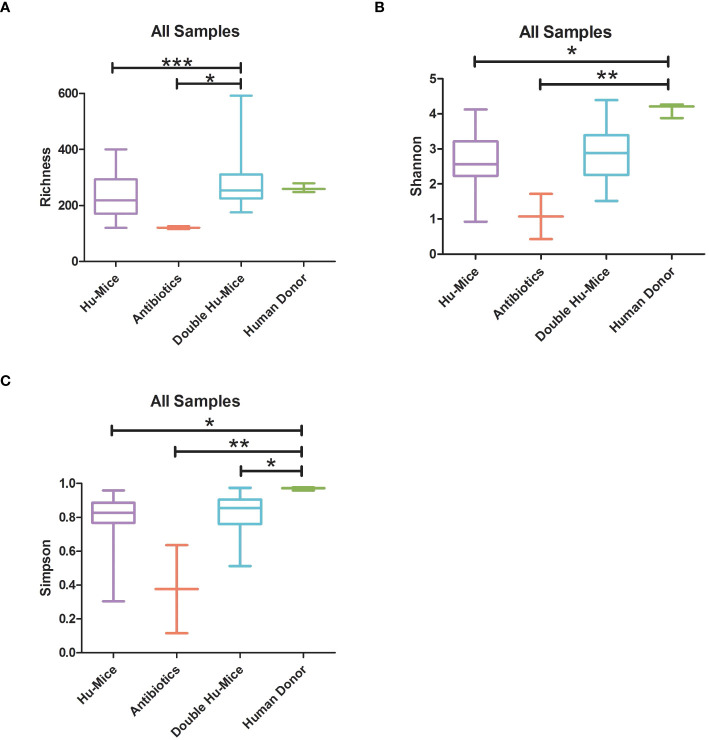
The gut microbiomes of double hu-mice had increased alpha diversity measures compared to that of pre-treatment or untreated control hu-mice. Alpha diversity indices Species richness **(A)**, Shannon index **(B)**, and Simpson index **(C)** between untreated control hu-mice, antibiotic-treated, double hu-mice, and human donor. Data are shown for pre-treatment or untreated control hu-mice (Hu-Mice), antibiotic treated mice (Antibiotics), double hu-mice (Double Hu-Mice), and human donor fecal samples (Human Donor). *, **, and *** indicate significant differences with p < 0.05, p < 0.01, and p < 0.001, respectively.

Since the highly immunodeficient NSG mice used for generating hu-mice were raised under SPF conditions with limited exposure to outside sources of microbes, we hypothesized that successful engraftment of donor microbiota would significantly increase the gut microbial diversity of double hu-mice compared to the hu-mice. We tested this hypothesis and found that the gut microbial diversity of double hu-mice with a functional immune system significantly increased to similar levels observed in our human donor samples versus the hu-mice. Several alpha diversity measurements confirmed that hu-mice had very low measures of alpha diversity compared to our human donor samples (**Figure 3**). However, after engraftment, double hu-mice had increased species richness compared to hu-mice (P < 0.001) and did not differ significantly from the human donor samples (**Figure 3A**). Further, the Shannon index values of the human donor samples were significantly higher than hu-mice (P < 0.05) but were not significantly different from the double hu-mice samples. Double hu-mice had increased Simpson index values compared to hu-mice, but the human donor samples were significantly higher than both the double hu-mice (P < 0.05) and hu-mice (P < 0.05). As expected, samples taken during antibiotic treatment had the lowest measures of all three diversity metrics evaluated. Alpha diversity metrics were also measured based on the human donor sample engrafted (**Supplementary Figure S1**). Double hu-mice engrafted after 14 days of antibiotics (donor 65 cohorts 1 & 2, donor mix) had higher levels of alpha diversity compared to double hu-mice engrafted after only seven days of antibiotics (donors 74 & 82). Overall, double hu-mice had increased alpha diversity and were more similar to that observed in the human donor samples compared to hu-mice. The shorter antibiotic treatment duration (seven days versus 14 days) was associated with lower alpha diversity measurements in double hu-mice engrafted with human donors 74 or 82.

### 3.3 The relative abundance of gut bacteria in double hu-mice was similar to that found in human donor samples

**Figure 4:**
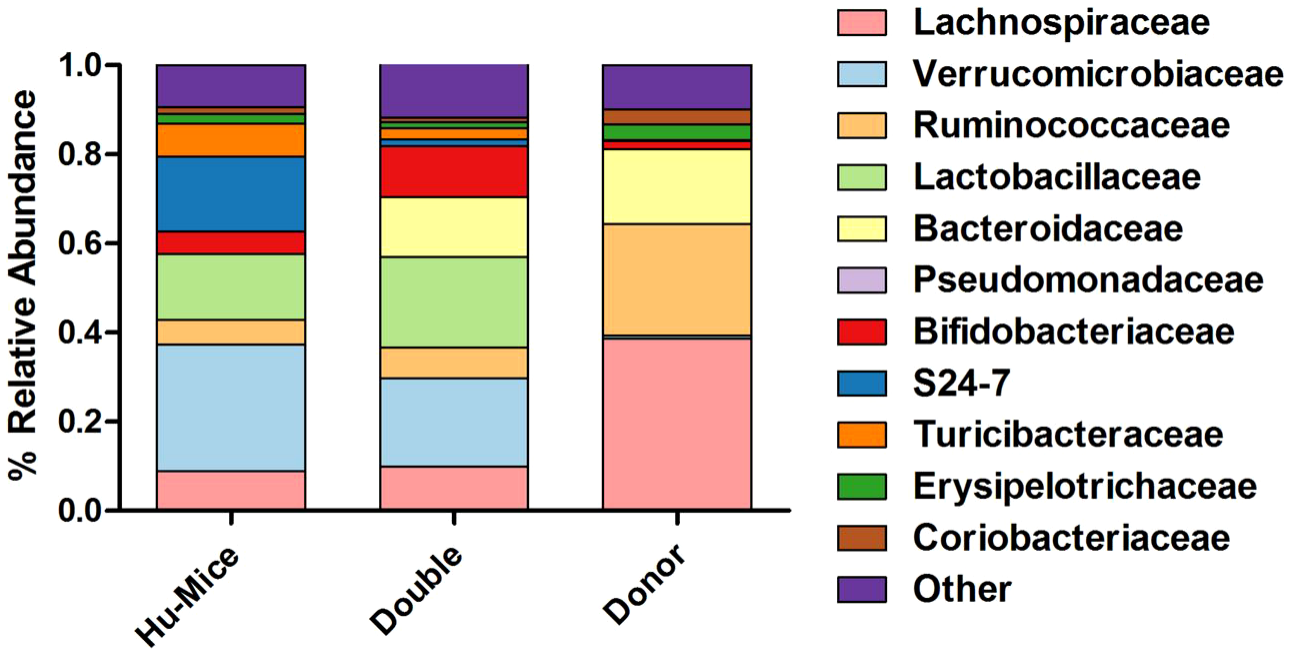
Comparison of relative abundance of taxa grouped by family. The 11 most abundant taxa by relative percent abundance grouped by family are shown for pre-treatment and untreated control hu-mice (Hu-Mice), double hu-mice (Double), and human fecal donor samples (Donor).

We next hypothesized that successful engraftment of human gut microbiota would result in more taxonomic similarities between the human donor gut microbiome profiles and double hu-mice versus hu-mice. Several differences were observed in the relative abundances of double hu-mice based on the length of antibiotic treatment, the human fecal donor sample engrafted, and mouse cohort (**Supplementary Figure S2**). At the phylum level, both double hu-mice and hu-mice samples were largely represented by *Actinobacteria*, *Bacteroidetes*, *Firmicutes*, and *Verrucomicrobia*. Notably, we found that hu-mice had high proportions of *Verrucomicrobia* that remained high even after antibiotic treatment and engraftment with human donor samples that had low abundances of *Verrucomicrobia*. At the family level, hu-mice samples had a higher relative abundance of *S24-7* than double hu-mice and human donor samples (**Figure 4**). Both hu-mice and double hu-mice samples had higher relative abundances of *Lactobacillaceae* and *Verrucomicrobiaceae* compared to human donor samples. The human donor and double hu-mice samples had higher relative abundances of *Bacteroidaceae* than hu-mice samples while *Lachnospiraceae* and *Ruminococcaceae* were relatively high in abundance in donor versus hu-mice and double hu-mice.

We next compared the taxonomic differences among microbiomes using Kruskal-Wallis testing with FDR adjusted P values below 0.05 (**Supplementary Table S1**). We found 195 significant differences between the hu-mice and human donor samples and 170 significant differences between hu-mice and double hu-mice. However, only 108 significant differences were observed between double hu-mice and human donor samples. Both the human donor and double hu-mice samples had significantly higher relative abundances of the family *Bacteroidaceae*, while hu-mice had a significantly higher relative abundance of the family *S24-7*. Human donor samples had a higher relative abundance of the phylum *Firmicutes* compared to both hu-mice and double hu-mice. Human donor samples had lower relative abundances of the class *Bacilli* and family *Lactobacillaceae* compared to both hu-mice and double hu-mice. Human donor samples also had a higher relative abundance of the class *Clostridia* than both hu-mice and double hu-mice samples (**Supplementary Figure S2**). Significant differences found within this class included the family *Lachnospiraceae*, genus *Blautia*, genus *Roseburia*, family *Ruminococcaceae*, genus *Ruminococcus*, and species *Faecalibacterium prausnitzii*. Some of these taxa did increase in abundance in double hu-mice as compared to hu-mice, as observed with significant differences in the genus *Blautia* and genus *Ruminococcaceae*. Levels of *Akkermansia muciniphila* were significantly higher in hu-mice and double hu-mice samples compared to human donor samples. However, double hu-mice had significantly less relative abundance of this species than hu-mice samples (**Supplementary Table S1**).

The increased similarity observed in double hu-mice and human donor gut microbiome profiles was driven by changes in the relative abundance of many taxa (**Supplementary Figure S3**). We categorized 70 bacterial taxa that had significantly higher relative abundance (Kruskal-Wallis testing with FDR adjusted P values below 0.05) in both human donor and double hu-mice samples compared to hu-mice. Further, 59 of the 70 higher abundance taxa were not significantly different between the human donor and double hu-mice samples. The largest changes were observed in members of *Bacteroidaceae* and *Blautia*. Additionally, increased abundance was observed in members of *Coriobacteriaceae*, [*Barnesiellaceae*], [*Odoribacteraceae*], *Porphyromonadaceae*, *Christensenellaceae*, *Eubacteriaceae, Lachnospiraceae, Ruminococcaceae, Veillonellaceae*, and *Erysipelotrichaceae*. *Proteobacteria* was also increased in human donor and double hu-mice compared to hu-mice, including members of *Burkholderiales, Desulfovibrionales*, and *Enterobacteriales.* We also identified seven bacterial taxa that had significantly lower relative abundance in both human donor and double hu-mice samples compared to hu-mice. All seven lower abundance taxa were not significantly different between the human donor and double hu-mice samples and belonged to *Coriobacteriaceae, S24-7*, and *Erysipelotrichaceae*. Altogether, changes in the relative abundance of many taxa drove the increased similarity between double hu-mice and human donor gut microbiome profiles.

As shown previously, double hu-mice had increased species richness compared to hu-mice. Part of this increased diversity may be derived from the engraftment of donor specific bacteria, or bacteria that were not found to be present in any pre-treatment or control hu-mice but were present in at least one human donor. We found 28 examples of donor specific bacteria that were transferred into double hu-mice across different taxa levels (**Supplementary Figure S4)**. The engrafted bacteria included members of *Bifidobacterium, Collinsella, Butyricimonas, Bacteroides, Streptococcus, Christensenellaceae, Eubacteriaceae, Anaerofustis, Pseudoramibacter Eubacterium, [Ruminococcus], Blautia, Coprococcus, Peptococcaceae*, [*Eubacterium*], and *cc-115*. The engraftment of these bacteria matched closely with the profiles of the human donor sample for each double hu-mice cohort. These results demonstrate that some of the increased diversity observed in double hu-mice is due to the engraftment of donor specific bacteria.

**Figure 5:**
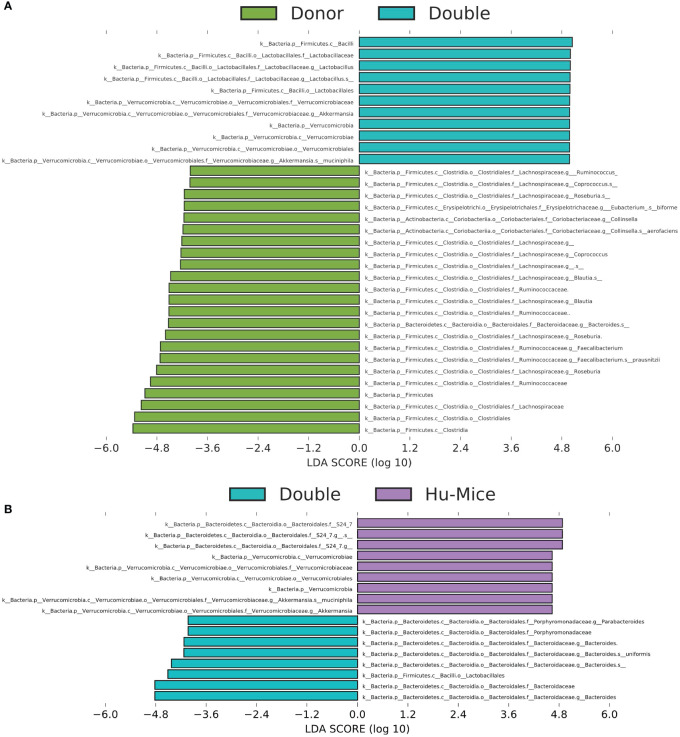
Engraftment of human fecal donor bacteria in double hu-mice as shown by LEfSe. All significant features with a linear discriminant analysis (LDA) score > 4.0 between human fecal donor samples (Donor) and double hu-mice (Double) **(A)** and between double hu-mice (Double) and pre-treatment or untreated control hu-mice (Hu-Mice) **(B)**.

To further determine differences in relative abundances among microbiomes from the various treatments, we used Linear discriminant analysis Effect Size (LEfSe) with a P value < 0.05 and LDA score > 2 (**Supplementary Table S2**) (Segata et al., 2011). Taxa with LDA scores higher than four were plotted to show significant differences between double hu-mice and human donor samples and double hu-mice and hu-mice samples. Human donor samples were associated with higher relative abundances of several types of *Clostridia*, including *Lachnospiraceae*, *Blautia*, *Coprococcus*, *Roseburia*, *Facalibacterium*, and *Ruminococcus* compared to double hu-mice, while double hu-mice samples were associated with *Lactobacillus* and *Akkermansia muciniphila* (**Figure 5A**). Double hu-mice were associated with higher relative abundances of *Bacteroides* and several types of *Clostridia*, including *Blautia*, *Coprococcus*, and *Ruminococcaceae*, while hu-mice samples were associated with *S24-7* and *Mogibacteriaceae* (**Figure 5B**, **Supplementary Table S2**). These findings demonstrate that certain taxa, like members of *Bacteroides*, readily engrafted double hu-mice while others such as *Clostridia* were more difficult to transplant. Furthermore, several species such as those found in the phylum *Verrucomicrobia* were highly prevalent in hu-mice, and antibiotic treatment followed by fecal transplant did not fully diminish or replace this population based on relative abundances. Altogether, these results demonstrate that engraftment of human donor samples significantly changed the taxonomic profile of double hu-mice and that their human-like gut microbiomes were statistically more similar to human donor profiles compared to hu-mice.

### 3.4 Stable engraftment of a human-like gut microbiome in double hu-mice

**Figure 6:**
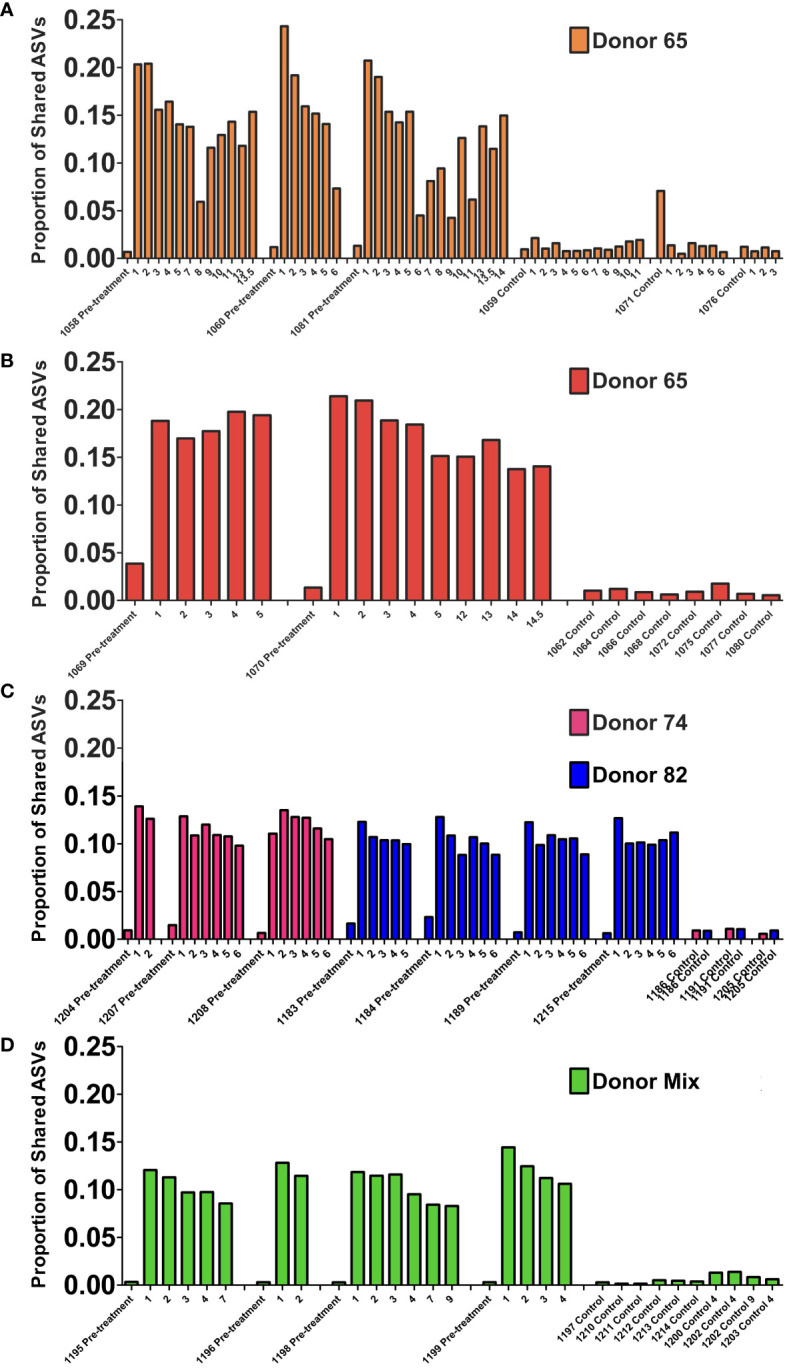
Engraftment and stability of a human-like gut microbiome in double hu-mice as determined by shared amplicon sequence variants (ASVs). Proportion of shared ASVs with the human donor from double hu-mice created using fecal material from human Donor 65, first cohort **(A)**; Donor 65, second cohort **(B)**; Donor 74 or Donor 82 **(C)**; and a mixture of all three donors **(D)**. On the x-axis, individual mouse IDs precede the Pre-treatment or Control designations followed by the number of weeks post fecal transplant.

To evaluate the stability of the engrafted human-like gut microbiome in double hu-mice after fecal transplant, the proportion of shared amplicon sequence variants (ASVs) with the human donor sample was calculated. After engraftment, double hu-mice had increased proportions of shared ASVs with their respective human donor samples, and those proportions remained higher than pre-treatment and control levels for the duration of the study. The first cohort of double hu-mice engrafted with human donor 65 had an average shared ASV proportion of 13.70% after transplant, while the pre-treatment samples had 1.06% and control samples levels had 1.42% (**Figure 6A**). This increased proportion of shared ASVs was maintained for the duration of the study, up to 14 weeks post-transplant (WPT). A second cohort of double hu-mice engrafted with human donor 65 was created, and a similar increase in the proportion of shared ASVs was observed (**Figure 6B**). The average proportion of shared ASVs was 17.65% post-transplant, while the pre-treatment samples had 2.60% and the control samples had 0.96%. This increased proportion of shared ASVs was maintained for the duration of the study, up to 14.5 WPT. In the case of the cohort treated with seven days of antibiotics prior to fecal transplant, the average shared ASVs proportions were 11.85% and 10.56% for double hu-mice mice engrafted with human donors 74 and 85, respectively, while the percentages of shared ASVs in pre-treatment and controls were found to be 1.02% and 0.86%, respectively, in cohort 74 and 1.33% and 0.95%, respectively, in cohort 82 (**Figure 6C**). For the double hu-mice transplanted with the mixture of all three human donors, the average shared ASV proportion was 10.91% after transplant, while the pre-treatment samples had 0.31% and the control samples had 0.61% (**Figure 6D**). Altogether, these results suggest a portion of donor fecal microbiota successfully engrafted in our double hu-mice and remained stable for the duration of the study.

**Figure 7:**
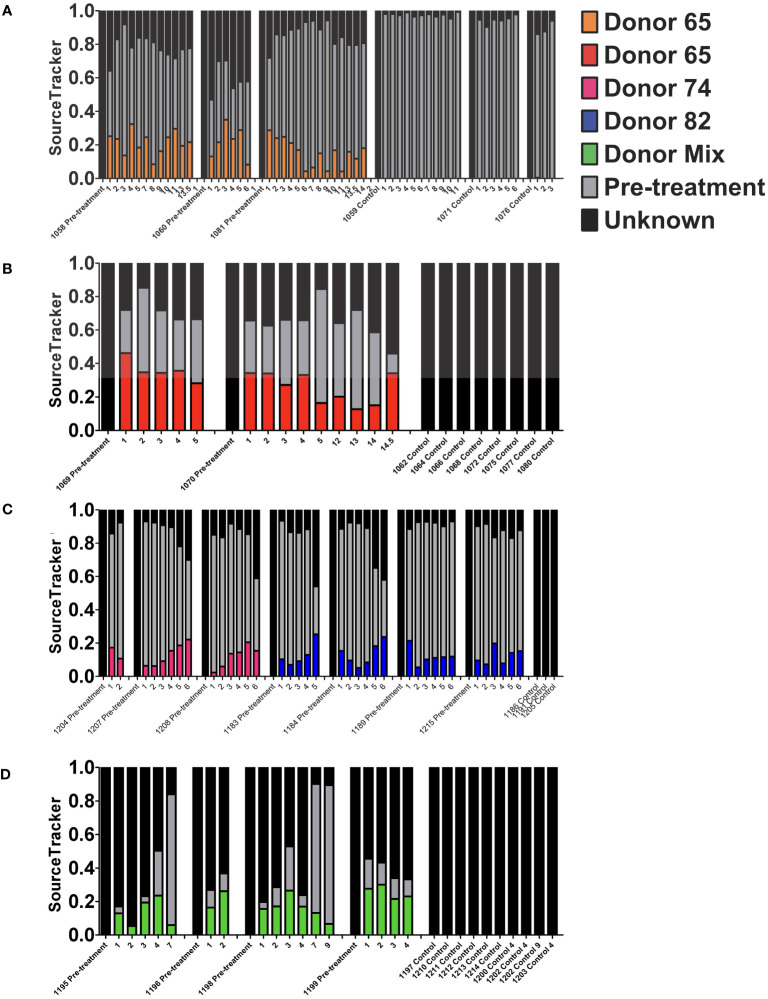
Stability of the engrafted human-like gut microbiome in double hu-mice as determined by SourceTracker. Contributions of the human fecal donor sample and pre-treatment sample in double hu-mice created using fecal material from Donor 65, first cohort **(A)**; Donor 65, second cohort **(B)**; Donor 74 or 82 **(C)**; and a mixture of all three donors **(D)**. On the x-axis, individual mouse IDs precede the Pre-treatment or Control designations followed by the number of weeks post fecal transplant.

To further evaluate the stability of the engrafted human-like microbiome after transplant into double hu-mice, the contributions of the human donor and pre-treatment sample to the post-transplant samples were determined using SourceTracker (Knights et al., 2011). The first cohort of mice transplanted with human donor 65 had an average donor contribution percentage of 18.76% after transplant, while the pre-treatment samples had 0.00% and the control samples had 0.05% (**Figure 7A**). At the final time point collected at 14 WPT, the donor contribution was consistent at 18.10%. The second cohort of mice transplanted with human donor 65 had an average donor contribution percentage of 29.01% after transplant, while the pre-treatment and controls samples had no donor contribution (**Figure 7B**). The donor contribution percentage was 34.10% at 14.5 WPT, thus demonstrating the stable engraftment of donor bacteria. Double hu-mice engrafted with donor 74 had an average donor contribution percentage of 12.71% after transplant, while the pre-treatment samples had 0.00% and the control samples had 0.00% (**Figure 7C**). At the final time point collected at 6 WPT, the average donor contribution was 18.75%.

The SourceTracker algorithm was unable to clearly distinguish donor 82 contributions as evidenced by both pre-treatment and control samples being assigned high human donor contribution percentages. Mice transplanted with human donor 82 had an average donor contribution percentage of 10.18% after transplant, while the pre-treatment samples had 11.95% and the control samples had 20.62% (**Figure 7C**). SourceTracker also assigned very high donor contribution percentages to the pre-treatment and control samples in the study of double hu-mice transplanted with a mixture of all three human donors. Mice transplanted with the mixture of all three human donors had an average donor contribution percentage of 14.88% after transplant, while the pre-treatment samples had 21.04% and the control samples had 15.89% (**Figure 7D**).

The high donor contribution percentages of donor 82 to the pre-treatment and control samples originated from an ASV with taxonomic assignment to *Akkermansia muciniphila*. This ASV was highly abundant in both hu-mice and double hu-mice and was much more prevalent in human donor 82 and the mixed donor sample compared to donors 65 or 74. To get a more accurate account of the stability of the human-like gut microbiome in post-transplant samples, we removed this ASV that was resulting in false positive donor contributions and once again used SourceTracker (**Supplementary Figure S5**). After the removal of the *A. muciniphila* ASV, the double hu-mice engrafted with human donor 82 had an average donor contribution percentage of 12.64% after transplant and double hu-mice engrafted with the mixture of all three human donors had an average donor contribution percentage of 18.11% after transplant, while all pre-treatment control samples were at 0.00%. Taken together, the results from shared ASVs and SourceTracker analyses, demonstrate that double hu-mice had a stable human-like gut microbiome for the duration of the study, extending up to 14.5 weeks post-transplant.

### 3.5 Increased human-like predicted metagenome functional content in double hu-mice

**Figure 8:**
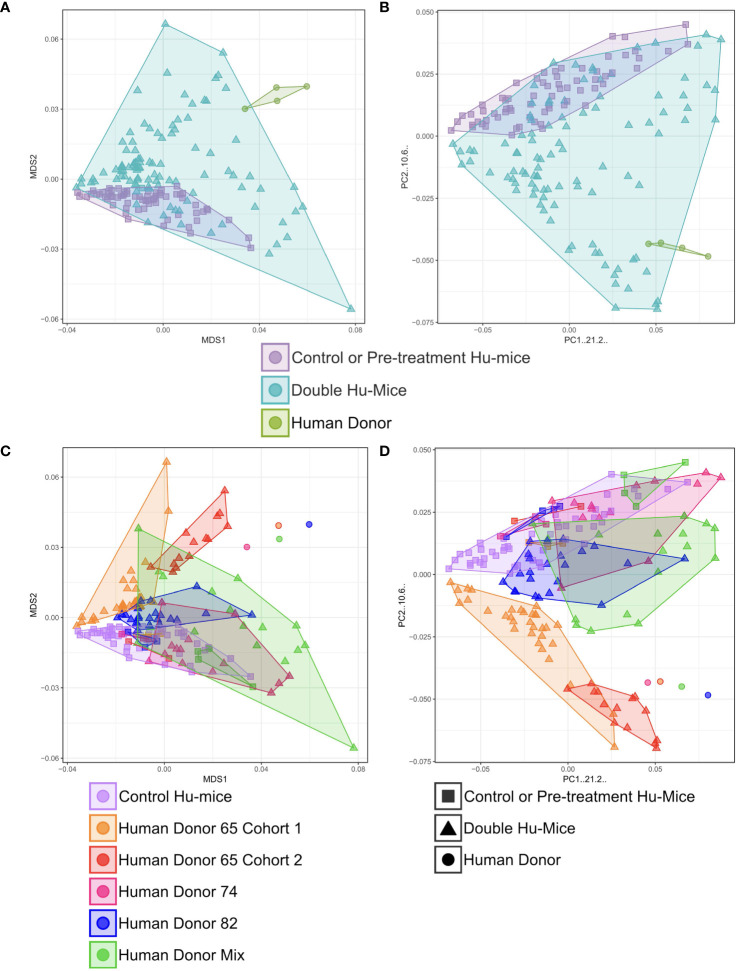
Increased human-like predicted metagenome functional content in double hu-mice. Rarified KEGG Orthology (KO) molecular functions were used to calculate Bray-Curtis dissimilarity after performing square-root transformations on the data. Non-metric multidimensional scaling (NMDS) or principal coordinates analysis (PCoA) ordination was used to create the coordinates for plotting the data. Double hu-mice clustered distinctly from the human fecal donor samples and pre-treatment or untreated control hu-mice in NMDS plot **(A)** and in PCoA plot **(B)**. Human donor specific functional profiles of double hu-mice in NMDS plot **(C)** and in PCoA plot **(D)**.

In addition to evaluating microbiome classification, we also sought to assess the functional capacity of the microbiomes in double hu-mice. PICRUSt was used to predict the metagenome functional content from the 16S rRNA data after ASV inference (Langille et al., 2013), and the predicted KO features were graphed using both NMDS and PCoA plots. Many of the double hu-mice samples clustered closer to the human donor samples than the hu-mice samples (**Figures 8A, B**). When color-coded by donor and cohort, the microbiomes that clustered closest to the human donor samples belonged to mice from the second cohort of double hu-mice generated by engrafting bacteria from human donor 65 (**Figures 8C, D**). Similarly, several samples from the other double hu-mice cohorts also separated themselves from the hu-mice cluster and were closer to the human donor samples. (**Figures 8C, D**).

We next tested for differences of each predicted KO feature among the three different groups of mice (**Supplementary Table S3**). In total, there were 4,513 non-zero predicted KO features. Using Kruskal-Wallis testing and an FDR adjusted p-value of < 0.05, we found 35.54% (1,604/4,513) significantly different predicted KO features between hu-mice and human donor samples. There were 39.09% (1,764/4,513) significantly different predicted KO features between double hu-mice and hu-mice samples. However, when we compared double hu-mice and the human donor samples, there were only 1.35% (61/4,513) significantly different predicted KO features. To clarify what functional aspects were missing from the double hu-mice gut microbiomes, we determined the predicted ASV contribution for each of the 61 significantly different KO features between the double hu-mice and human donor samples (**Supplementary Table S4**). This analysis provided insight into which functionally significant bacteria were not successfully engrafted from the human donor samples to the double hu-mice. A total of 95 ASVs with 73 unique taxonomic assignments were found to contribute to the 61 significantly different KO features (**Supplementary Figure S6**). Family level taxa with the highest levels of contribution included *Actinomycetaceae* (10.46%), *Bifidobacteriaceae* (8.76%), *Streptococcaceae* (20.74%), *Lachnospiraceae* (23.95%), and *Peptococcaceae* (8.66%). *Bifidobacterium adolescentis* was the highest contributing species (7.85%), and the highest contributing ASV (13.01%) had the taxonomic assignment of an unclassified *Streptococcus* species. Collectively, these analyses demonstrate that the predicted functional capacity of double hu-mice is more similar to human donor samples compared to hu-mice.

## 6 Discussion

Currently, humanized mice with human-like immune systems, yet devoid of human gut microbiota, are extensively employed to investigate the immune responses to human-specific pathogens. However, the absence of human gut microbiota may significantly compromise the translational relevance of these mouse model studies to humans, given the critical role gut microbiota play in immune response. Therefore, this study aimed to characterize the engrafted human fecal microbiota in our double humanized mice, which possess both a functional human immune system and a human microbiome, by comparing them to the currently used humanized mouse as control that has solely a human immune system. Furthermore, we compared engrafted human microbiota with donor samples and evaluated its stability.

We found that double hu-mice have a gut microbiome profile similar to that of human donor samples. Our approach created reproducible and donor-specific human-like gut microbiomes across multiple cohorts of double hu-mice. Further, we showed that double hu-mice had increased measures of diversity and increased functional capacity compared to hu-mice. The engrafted human-like gut microbiomes were also stable for the duration of the study, extending up to 14.5 weeks post-transplant. Importantly, we also demonstrated that the predicted functional capacity of double hu-mice is more similar to the human donor samples than hu-mice.

One of the most significant findings of this study was that the double hu-mice had gut microbiome profiles that were unique to the human donor engrafted. Each of the four different donor samples resulted in a distinct population of double hu-mice resembling the human donor. This observation has several practical implications, particularly within personalized medicine and translational research domains. Patient-derived microbiotas can be used as donor microbiotas, and this mouse model can be utilized to study personalized responses of patient microbiomes to drug regimes, therapies, or dietary interventions. Additionally, this double hu-mice model could present a viable avenue to explore the mechanistic underpinnings of patients’ pathological states, while concurrently offering a platform to understand the interplay between the patient’s gut microbiome and the etiology of disease.

We found that double hu-mice had increased measures of alpha diversity compared to hu-mice. Several studies have highlighted the importance of microbiome diversity within the gut and have linked low gut microbiome diversity with several disease conditions (Chang et al., 2008; Willing et al., 2010). While not all low diversity conditions are detrimental, specifically when there is enrichment of potentially beneficial bacteria through prebiotic or probiotic treatments, the low pre-existing diversity found within the hu-mice was far below the levels observed in our human donor samples. After engraftment, the double hu-mice had increased levels of alpha diversity and maybe more importantly, had increased predicted functional capacities. This increased diversity may also offer a more realistic gut environment as it allows for diverse reciprocal interactions with the engrafted human immune system.

We also found that the engrafted human-like microbiome was very stable in our model for the length of the study, up to 14.5 weeks after transplant. We used several methods to determine the engraftment level and stability of the gut microbiome after transplant and found no reversion to the pre-existing murine profile. This stability allows for the longitudinal study of the role of the gut microbiome in many human diseases, such as HIV-1 and cancer. One outstanding question is whether the unique presence of human immune cells plays a role in stabilizing or enhancing engraftment of the human-like gut microbiome in our model compared to other non-humanized mouse models. Our data showed no reversion to the pre-existing murine gut microbiome profile, perhaps due to some enhanced stability or selection by the reconstituted human immune system. Further studies are needed to determine the relationship between the engrafted gut microbiome and the human immune system.

Many different methods and antibiotic regimens have been used for preconditioning of mice prior to fecal transplantation (Reikvam et al., 2011; Ericsson et al., 2017; Staley et al., 2017). Different combinations and durations of antibiotic treatments may increase the efficiency of the fecal transplant into the host (Staley et al., 2017). While the combination of metronidazole, ampicillin, neomycin, and vancomycin is widely used due to its broad spectrum of bacterial targets, the best methods are still being investigated. We found that the very rigorous method of gavaging antibiotics twice daily for 14 days used by Hintze et al. was too invasive for our NSG hu-mice and resulted in increased mortality (Hintze et al., 2014). Our study suggests that administrating broad-spectrum antibiotics in drinking water for a duration of 14 days represents an effective approach for preconditioning NSG hu-mice prior to fecal transplantation, thus ensuring improved health and survival outcomes compared to more invasive regimens for generating double hu-mice.

Other important practical implications of our mouse model are the logistics and technical requirements. Although the germ-free mice model is considered a gold standard method for FMT studies and may exhibit better engraftment of most donor microbiota, germ-free animals require gnotobiotic facilities and expensive, specialized equipment (Kennedy et al., 2018; Le Roy et al., 2018). However, our double hu-mice have the advantage of requiring only SPF housing conditions, which are widely available and less costly compared to germ-free facilities. Our method also does not perturb the complex surgical procedures in generating BLT hu-mice, as it does not mandate the creation of a completely germ-free environment. Furthermore, our method of generating a double hu-mice model can be adapted to the neonatal intrahepatic hCD34+ hematopoietic stem progenitor cell (HSPC) method of humanization, which is a comparatively simpler process compared to the surgical approach integral to the BLT humanization procedure.

Our double hu-mice do not exhibit complete engraftment of donor fecal microbiota, which can be attributed to several hypothesized factors. There are major differences between the human and mouse digestive tract, including structure, function, and pH (Nguyen et al., 2015). Our mice are not germ-free to begin with, and the broad-spectrum antibiotic treatment can only reduce the prevalence of pre-existing murine gut bacteria. There were several key differences in the reconstituted mice compared to the human donors. Double hu-mice had significantly lower levels of several types of *Clostridia* including *Lachnospiraceae*, *Blautia*, *Coprococcus*, *Roseburia*, *Faecalibacterium*, and *Ruminococcus* compared to human donor samples. Many of these bacteria are well documented to be difficult to reconstitute within germ-free and SPF mouse models (Turnbaugh et al., 2009; Wos-Oxley et al., 2012; Hintze et al., 2014). Similar to fecal transplants in humans designed to treat *C. difficile* infections, the engrafted human-like gut microbiome in our double hu-mice is the result of a combination of host, donor, and environmental bacteria (Smillie et al., 2011). Despite these previously known limitations, our double hu-mice model reproducibly results in a donor-specific, stable, human-like gut microbiome in the presence of a human immune system.

## 7 Conclusion

Here, we describe the successful and stable engraftment of human fecal microbiomes into immunodeficient NSG mice surgically implanted with a functional human immune system to create double hu-mice with human donor-specific human gut microbiomes. This mouse model is a useful platform to study the impact of pathogen and dietary intervention on human health in the presence of both a functional human immune system and a human-like gut microbiota with minimal logistics and technical requirements.

## Data availability statement

The original contributions presented in the study are publicly available. This data can be found here: NCBI SRA, accession PRJNA507247.

## Ethics statement

The studies involving humans were approved by the Scientific Research Oversight Committee (SROC) at the University of Nebraska-Lincoln (UNL) (SROC# 2016-1-002). The studies were conducted in accordance with the local legislation and institutional requirements. The human samples used in this study were acquired from Advanced Bioscience Resources (Alameda, CA). Written informed consent for participation was not required from the participants or the participants’ legal guardians/next of kin in accordance with the national legislation and institutional requirements. The animal study was conducted in accordance with Institutional Animal Care and Research Committee (IACUC)-approved protocols at the University of Nebraska-Lincoln (UNL). The IACUC at UNL has approved two protocols related to generating and using humanized BLT (hu-BLT) mice, including double hu-mice.

## Author contributions

LD: Conceptualization, Data curation, Formal analysis, Investigation, Methodology, Software, Validation, Visualization, Writing – original draft, Writing – review & editing. SL: Writing – review & editing. AR-T: Supervision, Writing – review & editing. QL: Conceptualization, Funding acquisition, Project administration, Resources, Supervision, Visualization, Writing – review & editing.
