# Supplementary material for: Characterization of double humanized BLT-mice with stable engraftment of a human gut bacterial microbiome
Source: Front Microbiomes. 2024 Jul 4;3:1404353. doi: 10.3389/frmbi.2024.1404353 (PMC12993508; doi:10.3389/frmbi.2024.1404353)
Supplement: Supplementary file 5 [file DataSheet_1.docx]

**Characterization of Double Humanized BLT-mice with Stable Engraftment of a Human Gut Bacterial Microbiome**

Lance Daharsh^1,2#+^, Saroj Chandra Lohani ^1,2+^, Amanda E. Ramer-Tait^3,4^, Qingsheng Li^1,2*^

Authors’ affiliations:

^1^School of Biological Sciences, University of Nebraska-Lincoln, Lincoln, NE, United States

^2^Nebraska Center for Virology, University of Nebraska-Lincoln, Lincoln, NE, United States

^3^Department of Food Science and Technology, University of Nebraska-Lincoln, Lincoln, NE, United States

^4^Nebraska Food for Health Center, University of Nebraska-Lincoln, Lincoln, NE, United States

#Current address: Genvax Technologies, Ames, IA, United States

+These authors contributed equally to this work

*Address correspondence to Qingsheng Li, [qli@unl.edu](mailto:qli@unl.edu)

Figure S1: Alpha diversity index (Species Richness, Shannon, and Simpson) of hu-mice, double hu-mice cohort, and human donor samples. *, **, and *** indicate significant differences with P < 0.05, P < 0.01, and P < 0.001, respectively.

Figure S2: Relative abundance of pre-treatment, hu-mice, human fecal donor, and double hu-mice cohort samples at various taxonomic level.

Figure S3: Change in the relative abundance of various taxa in control or pre-treatment hu-mice, Double hu-mice, and human donor.

Figure S4: Donor specific taxa that were transferred into double hu-mice.

Figure S5: Contributions of the human fecal donor sample and pre-treatment sample in double hu-mice created using fecal material from donor 82 and donor mix after removing the ASV that was resulting false positive donor contributions.

Figure S6: Taxa contributing significantly different KO features.
